# Pooling size sorted Malaise trap fractions to maximize taxon recovery with metabarcoding

**DOI:** 10.7717/peerj.12177

**Published:** 2021-10-05

**Authors:** Vasco Elbrecht, Sarah J. Bourlat, Thomas Hörren, Angie Lindner, Adriana Mordente, Niklas W. Noll, Livia Schäffler, Martin Sorg, Vera M.A. Zizka

**Affiliations:** 1 Centre for Biodiversity Monitoring, Zoological Research Museum Alexander Koenig, Bonn, Germany; 2 SimplexDNA AG, Winterthur, Switzerland; 3 Entomological Society Krefeld, Krefeld, Germany

**Keywords:** Metabarcoding, Size sorting, Biodiversity, Monitoring, Pooling strategies, Insects

## Abstract

**Background:**

Small and rare specimens can remain undetected when metabarcoding is applied on bulk samples with a high specimen size heterogeneity. This is especially critical for Malaise trap samples, where most of the biodiversity is contributed by small taxa with low biomass. The separation of samples in different size fractions for downstream analysis is one possibility to increase detection of small and rare taxa. However, experiments systematically testing different size sorting approaches and subsequent proportional pooling of fractions are lacking, but would provide important information for the optimization of metabarcoding protocols. We set out to find a size sorting strategy for Malaise trap samples that maximizes taxonomic recovery but remains scalable and time efficient.

**Methods:**

Three Malaise trap samples were sorted into four size classes using dry sieving. Each fraction was homogenized and lysed. The corresponding lysates were pooled to simulate unsorted samples. Pooling was additionally conducted in equal proportions and in four different proportions enriching the small size fraction of samples. DNA from the individual size classes as well as the pooled fractions was extracted and metabarcoded using the FwhF2 and Fol-degen-rev primer set. Additionally, alternative wet sieving strategies were explored.

**Results:**

The small size fractions harboured the highest diversity and were best represented when pooling in favour of small specimens. Metabarcoding of unsorted samples decreases taxon recovery compared to size sorted samples. A size separation into only two fractions (below 4 mm and above) can double taxon recovery compared to not size sorting. However, increasing the sequencing depth 3- to 4-fold can also increase taxon recovery to levels comparable with size sorting, but remains biased towards biomass rich taxa in the sample.

**Conclusion:**

We demonstrate that size fractionation of Malaise trap bulk samples can increase taxon recovery. While results show distinct patterns, the lack of statistical support due to the limited number of samples processed is a limitation. Due to increased speed and lower risk of cross-contamination as well as specimen damage we recommend wet sieving and proportional pooling of the lysates in favour of the small size fraction (80–90% volume). However, for large-scale projects with time constraints, increasing sequencing depth is an alternative solution.

## Introduction

DNA metabarcoding is a useful approach to characterize arthropod communities. Instead of DNA barcoding individual specimens (Hebert et al., 2003), DNA is usually extracted from homogenized bulk samples (Zizka, Geiger & Leese, 2020; Mata et al., 2020; Turunen et al., 2021; Hardulak et al., 2020). A barcoding marker is amplified for the whole community and sequenced with high throughput sequencing. This allows for the identification of entire communities (Ji et al., 2013), often at species level resolution, depending on marker choice and reference database completeness (Leray et al., 2019; Weigand et al., 2019). However, DNA metabarcoding is affected by a large number of methodological biases preventing accurate specimen counts and often not all taxa in the sample are detected (Elbrecht et al., 2017; Piper et al., 2019). While there is a loose correlation between biomass and read abundance (Lamb et al., 2019), metabarcoding results are often skewed by primer bias (Elbrecht & Leese, 2015; Piñol et al., 2015; Krehenwinkel et al., 2017), mitochondrial copy number variation (Braukmann et al., 2019; Deagle et al., 2019), sequencing errors (Singer et al., 2019) and variation in specimen size, which can result in smaller taxa remaining undetected (Elbrecht, Peinert & Leese, 2017).

Arthropod bulk samples often show a wide range of specimen sizes (Aylagas et al., 2016). While imaginal stages of the same species have similar biomass, there can be substantial size variation between the life stages within species and of course between different species (Stork & Blackburn, 1993; Siemann, Tilman & Haarstad, 1996). Thus, when extracting DNA from bulk samples containing rare or small taxa with low biomass, those might be overshadowed by biomass rich taxa that contribute a large amount of DNA to the extraction. This natural variation can jeopardize the detection of small and rare specimens (Deagle et al., 2018), especially when taking PCR effects into account (Kelly, Shelton & Gallego, 2019). To maximize taxon detection in bulk samples showing substantial specimen biomass variation (Aylagas et al., 2016), size sorting might be required (Liu et al., 2020). Metabarcoding of marine arthropod bulk samples is commonly preceded by size fractioning of samples through sieving (Cowart et al., 2015; Leray & Knowlton, 2015; Wangensteen & Turon, 2017; Wangensteen et al., 2018). Size sorting has also been applied to freshwater macroinvertebrates (Elbrecht, Peinert & Leese, 2017; Carew, Coleman & Hoffmann, 2018; Zizka et al., 2019) and terrestrial arthropods (Ji et al., 2013; Creedy, Ng & Vogler, 2019; Hausmann et al., 2020). While the advantages of size sorting seem logical, the process can take a substantial amount of time if done manually (Elbrecht, Peinert & Leese, 2017) or even when using semi-automated methods like sieving. Additionally, while size sorting likely increases taxon recovery, it also generates sub samples that can either be pooled or sequenced individually. Regardless of the chosen size sorting and metabarcoding strategy, the amount of laboratory work and cost increases compared to the processing of a single metabarcoding bulk sample without size sorting. Thus, some authors suggest increasing sequencing depth instead of applying size sorting strategies, especially when size variation is limited (Creedy, Ng & Vogler, 2019; Elbrecht, Peinert & Leese, 2017). In fact, most non-marine studies do metabarcode complete bulk samples without prior size sorting (*e.g*. Hardulak et al., 2020; Hausmann et al., 2020; Steinke et al., 2020; Bush et al., 2020).

In this study, we explore the effects of size sorting Malaise trap samples through sieving into four size fractions to maximize taxon recovery. We thereby want to circumvent time consuming and therefore expensive manual sorting, which is not feasible for large-scale approaches. Furthermore, manual sorting and determination of samples is difficult to standardize and depends on the researcher performing the sorting. We therefore focus on reproducible sieving approaches including a standardized mesh size and a defined sieving time and amplitude. Prior to DNA extraction, we specifically test the effect of pooling lysates of each size fraction in different proportions. Each size fraction is also extracted and metabarcoded individually, giving us the opportunity to further validate different size sorting scenarios. The goal of this study is to find a standardized and scalable DNA metabarcoding strategy for Malaise trap samples, which also captures the diversity of small and rare specimens.

## Materials and Methods

### Samples and size sorting

Arthropods were collected on a meadow near Eschweiler, Germany (50.576708N, 6.730254E) using a Townes style Malaise trap (Ssymank et al., 2018). The trap was set up in the Natural Park “Hohes Venn” (calcareous grassland managed through grazing) with special permit from the nature conservation authority Euskirchen (reference number: 67.64.300/Pa). The trap was run with 80% denatured ethanol with 1% methyl ethyl ketone and samples were collected in 2017 from June 5th to June 26th (sample L1), from July 14th to August 11th (sample L2) and from May 16th to June 5th (sample L3). Samples were dried in an incubator (INCU Line ILS 6; VWR, Radnor, PA, USA) at 50 °C for 5 days. Samples were size sorted with a vibratory sieve shaker (AS 300 control; Retsch, Haan, Germany) using three stacked perforated plate sieves (305 mm in diameter) with 8, 4, and 2 mm diameter round holes respectively (Retsch, Haan, Germany). Sieving was performed for 5 min at 1.5 mm amplitude. Four size fractions (S < 2 mm, M > 2 mm, L > 4 mm, XL > 8 mm) were obtained. All specimens electrostatically attracted to the sieves were collected with tweezers and equipment was thoroughly cleaned with bleach between samples. Purified water was used for bleach removal and the equipment was subsequently UV irradiated for 5 min.

Size sorting of Malaise trap samples with the method outlined above takes 5–10 min, but removal of electrostatically charged specimens from the sieving equipment is time consuming and can take 2–3 h per sample. Thus, a second and third sieving method was explored (Fig. S1), but not sequenced due to time and financial constraints. Those alternative methods were tested on different samples than the dry sieving approach introduced in the previous section (L1–L3). Samples for the testing of alternative sieving approaches were collected in the nature protected area “Latumer Bruch”, Lohbruchweg, Krefeld, Germany (51.326701N, 6.632973E) from 18th to 29th of May 2019 (dry sieving “shaker”) and in a nature protected area near Vlotho, Germany (52.122705N, 8.785186E) from 19th of September to 3rd of October 2017 (wet sieving “lunch box”). For the second method, a dry sieving shaker was built. The shaker was constructed using one 500 ml and one 1 l sampling bottle (Kautex Textron GmbH & Co. KG, Bonn, Germany) which were connected with a metal mesh of either eight mm or four mm mesh size (Haver & Boecker OHG, Oelde, Germany) to obtain three size fractions (>8, >4, <4 mm). Dried specimens were separated into size classes by manually shaking the bottle for 2–3 min but subsequent specimen recovery was also affected by electrostatic charging from the plastic. As a third alternative size sorting approach, a four mm metal mesh (Haver & Boecker OHG, Oelde, Germany) was placed in a round glass container (IKEA365+; Ikea, Delft, Netherlands). The edges of the mesh were sealed with hot glue for better grip and stability within the glass dish. The sample was emptied onto the grid and fully immersed in 96% ethanol without prior drying. With repeated light shaking and pauses of 10 s, specimens could be separated into a small (<4 mm) and a large (>4 mm) size fraction. Specimens contained in both size fractions were dried and all specimens were measured and weighed to assess the effectiveness of this size sorting approach. Size measurements refer to the length of specimens as measured on a millimetre scale paper (excluding antennae and legs, Fig. S1).

### Tissue homogenization, lysate pooling and DNA extraction

**Figure 1:**
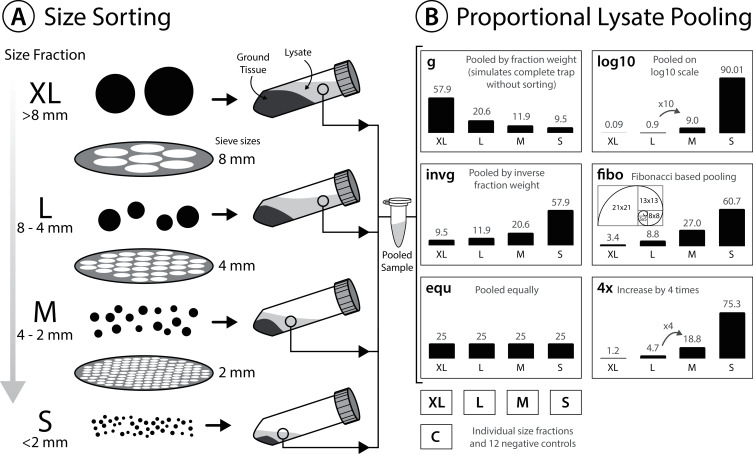
Experimental design testing the effectiveness of size sorting Malaise trap samples and pooling the fractions in different proportions. (A) Dried specimens are sieved into four different size classes, homogenized and lysed with a buffer amount corresponding to specimen weight in the respective fraction. (B) Lysate is incubated and pooled in different amounts. Grey numbers above the bars indicate the relative proportions (%). All six lysate pools were generated in duplicate, each individual size fraction (S, L, M, XL) was also extracted in duplicate, and nine negative controls were included.

To reduce electrostatic charging after dry sieving, sieved insects were moistened with 96% ethanol before transferring them into 30 ml grinding tubes (Nalgene; Thermo Fisher Scientific, Waltham, MA, USA). Specimens were dried again for up to 5 days at 50 °C in the oven (INCU Line ILS 6, VWR, Radnor, PA, USA). Homogenization was performed in grinding tubes with five mm diameter steel beads (Retsch, Haan, Germany), using a mixer mill (MM400; Retsch, Haan, Germany) for 2 min at 30 hz. Ground tissue from each size fraction was transferred to either 50 ml falcon tubes or 500 ml bottles and weighed. For tissue lysis, 10 ml ATL lysis buffer (Qiagen, Hilden, Germany) supplemented with 2% Proteinase K (20 mg/ml) was added per g of tissue. Ground tissue with lysis buffer was incubated overnight at 56 °C in a shaking incubator (INCU Line ILS 6; VWR, Radnor, PA, USA) and centrifuged for 5 min at 4,149 g (Mega Star 1.6; VWR, Radnor, PA, USA). Samples were centrifuged to pelletize solid tissue at the bottom of the tubes and to facilitate the pooling of lysates by pipetting the respective proportions of supernatant. Thereby, lysates of the four size fractions (S < 2 mm, M > 2 mm, L > 4 mm, XL > 8 mm) were pooled in volumes ranging from 0.18 to 200 µl of supernatant (see Fig. 1 & Table S1 for details of lysate volumes pooled for the different ratios). In total, 200 µl of pooled (or single fraction) lysate was used for each DNA extraction. Since the amount of extraction buffer added was proportional to the weight of tissue in the respective size fraction, we assumed the DNA concentration in each lysate to be the same. Thus, pooling lysate volumes of the four size fractions proportional to the tissue weight simulates an unsorted Malaise trap sample (L1_gA, L2_gA, L3_gA). The other schemes (shown in Fig. 1, Table S1) simulate different pooling strategies of size fractions to test the effects on taxon recovery. In addition, individual size fractions (S, M, L, XL) of all three samples were also processed and sequenced individually and used for later *in silico* analysis.

DNA was extracted from the lysate using a Qiagen DNeasy 96 well Blood & Tissue Kit (Qiagen, Hilden, Germany) following the manufacturer’s protocol with only one adjustment (2% proteinase K). DNA was eluted with 200 µl buffer AE (Qiagen, Hilden, Germany). The 96 well plate map with sample locations is available as supporting information (Fig. S2), with 27 wells being used for primer tests as part of a separate project. In nine negative controls only ATL lysis buffer (Qiagen, Hilden, Germany) and Proteinase K without tissue were used for extraction (DNeasy 96 well Blood & Tissue Kit; Qiagen, Hilden, Germany). The eluates obtained from these negative controls were also included in downstream amplicon and indexing PCR. DNA quality was examined *via* gel electrophoresis on a 0.5% agarose gel with 0.1% GelRed (10,000×; Biotium Inc., Fremont, CA, USA) and 1× TBE. One µl of GeneRuler 100 bp plus DNA ladder (Thermo Fisher Scientific, Waltham, MA, USA) and three µl of each DNA extract were loaded onto the gel.

### DNA metabarcoding

Sample metabarcoding was carried out for the six different pooling strategies of samples L1, L2 and L3 and also for each size fraction individually. An extraction replicate was included for each reaction. A two-step approach with standard illumina Nextera primers (San Diego, CA, USA) was used for dual tagging in PCR 2 (index PCR). All primers were obtained from Metabion International AG, Munich, Germany. PCR 1 (amplicon PCR) was carried out in a 96 well plate using reactions of 25 µl with one µl DNA (stock undiluted), 0.2 µM of each fwhF2 forward primer (Vamos, Elbrecht & Leese, 2017) and Fol-degen-rev reverse primer (Yu et al., 2012), 12.5 µl PCR Multiplex Plus buffer (Qiagen, Hilden, Germany) and 10.5 µl ddH_2_O (Sigma–Aldrich, St. Louis, MO, USA Sigma–Aldrich). Primers included an universal Nextera tail to facilitate sample tagging in PCR 2. The thermal cycler 2,720 from ABI was used and set with the initial denaturation at 95 °C for 5 min; 30 cycles of: 30 sec at 95 °C, 30 sec at 50 °C and 50 sec at 72 °C; and a final extension of 5 min at 72 °C. One µl of PCR product was used as a template for the second PCR (no cleanup applied, Elbrecht & Steinke, 2019). In the second PCR 12 times 8 Nextera (Illumina, San Diego, CA, USA) based primer combinations were used to attach eight bp dual indexes to individual samples as well as the tails needed for Illumina sequencing (see Fig. S2 for further information on adapter combinations used). The 25 µl reactions in the second PCR were composed of one µl PCR1 (undiluted), 0.4 µM of each primer (Nextera, Illumina, San Diego, CA, USA), 12.5 µl PCR Multiplex Plus buffer (Qiagen, Hilden, Germany) and 10.5 µl ddH_2_O. A higher concentration of primers (0.4 µM) was used in the second PCR than in the first PCR (0.2 µM) to achieve better DNA amplification. The same PCR cycling conditions as in the first PCR step were used, but the cycle number was reduced to 15. PCR success was checked on a 0.7% and 1% agarose gel for the first and second PCR, respectively. A higher agarose concentration allows a better separation of smaller bands *via* electrophoresis, thereby visualizing potentially problematic byproducts. Gels were composed as described above for the DNA quality check. PCR products were normalized using SequalPrep Normalization Plates (Thermo Fisher Scientific, Waltham, MA, USA; Harris et al., 2010) to obtain 25 ng product per reaction according to the manufacturer’s protocol. Normalization success was verified on a 1% agarose gel as described above. A total of 10 µl of each normalised sample library was pooled, and the final library was cleaned up twice using a left sided size selection with 0.76× and 0.65× SPRIselect (Beckman Coulter, CA, USA) to completely remove primer dimers (including negative controls). The final library concentration (2.01 ng/µl) was measured using the QuantiFluor dsDNA system (E2670; Promega, Madison, WI, USA) and a Quantus fluorometer (Promega, Madison, WI, USA). Cleanup success was verified by running samples on a Fragment Analyzer (Agilent Technologies, Santa Clara, CA, USA) using the HS NGS Fragment Kit (Agilent Technologies, Santa Clara, CA, USA). The Fragment Analyzer data were evaluated using the software PROSize 2.0. Sequencing was carried out by Starseq (Mainz, Germany) using a 600 cycle Illumina MiSeq Reagent Kit v3 and 5% PhiX spike in.

### Bioinformatic processing

Raw sequence data were delivered in blc format and converted to fastq files using bcl2fastq v2.20.0.422 and the option—create-fastq-for-index-reads. Sequence quality was verified using FastQC v0.11.9. Since there were ‘no call’ issues with the sequencing run, the base calls for each flow cell tile in each cycle were plotted and evaluated for problems using the PlotTiles function in JAMP v0.77 (github.com/VascoElbrecht/JAMP). No-call events were limited to the primer binding regions. Sequences were demultiplexed using deML setting exact matching (github commit dd87669 (Renaud et al., 2015)). Sequence data was processed using the JAMP pipeline that mostly relies on Vsearch v2.14.2 (Rognes et al., 2016) and Cutadapt v2.8 (Martin, 2011). Scripts are available in Script S1 (“JAMP_lysis_test_v3.R”). Paired-end merging of forward and reverse read was done using Vsearch with fastq_maxdiffpct=25 to maximize the number of reads merged. Primer sequences were removed from each sample using Cutadapt, allowing for 30% mismatches in the binding region, while retaining only sequences where both primers were successfully trimmed. Cutadapt was also used to remove sequences deviating more than 10 bp from expected amplicon length of 313 bp. Poor quality sequences with an expected error score above 1 (Edgar & Flyvbjerg, 2015) or containing N base calls were removed using Vsearch. All samples were pooled, dereplicated (minsize = 2) and clustered into Operational Taxonomic Units (OTUs) using Vsearch with cluster_smallmem and 97% similarity, followed by denovo chimera removal. Individual samples were dereplicated (retaining singletons) and mapped against the OTUs to generate an OTU table, using usearch_global with maxrejects = 256 and maxaccepts = 16. Potential false OTUs were flagged using LULU version 0.1.0 (Frøslev et al., 2017) and taxonomic assignment was conducted with https://www.gbif.org/tools/sequence-id. Subsequently, only flagged OTUs that shared the same taxonomy were merged. Flagged OTUs that were assigned to different taxonomies were analysed as separate molecular units for downstream analysis. Direct OTU clustering was used instead of denoising, to maximize the amount of sequences retained. Additionally, given the good coverage in the reference data, the removal of spurious OTUs with LULU gives a good confidence in OTU accuracy. For the comparison between size ratios OTU clustered data are sufficient and also represents species more closely than exact sequence variants (ESVs) which give a haplotype level resolution within species. Data were further cleaned up by subtracting the maximum read number of each OTU in the negative controls from all other samples (Elbrecht & Steinke, 2019). Additionally, all read counts with an abundance below 0.01% per sample were discarded as well as counts not present in both extraction replicates. Filtering all samples at 0.01% relative abundance ensures comparisons are done at the same sequencing depth, while reducing stochastic effects introduced by repeated rarefaction of the data. As downstream data analysis was performed with proportional read counts, filtering at 0.01% abundance is comparable to a sequencing depth of 10,000 sequences per sample. Additionally, since all reads were discarded that were not present in both extraction replicates with at least 0.01% abundance, the two replicates were combined for analysis. Since the GBIF database is clustered at 99% similarity, BOLDigger v1.1.3 (https://github.com/DominikBuchner/BOLDigger) was used to assign taxonomy to the OTU subset to improve taxonomic matching (setting “JAMP pipeline”).

### *In silico* analysis

**Figure 2:**
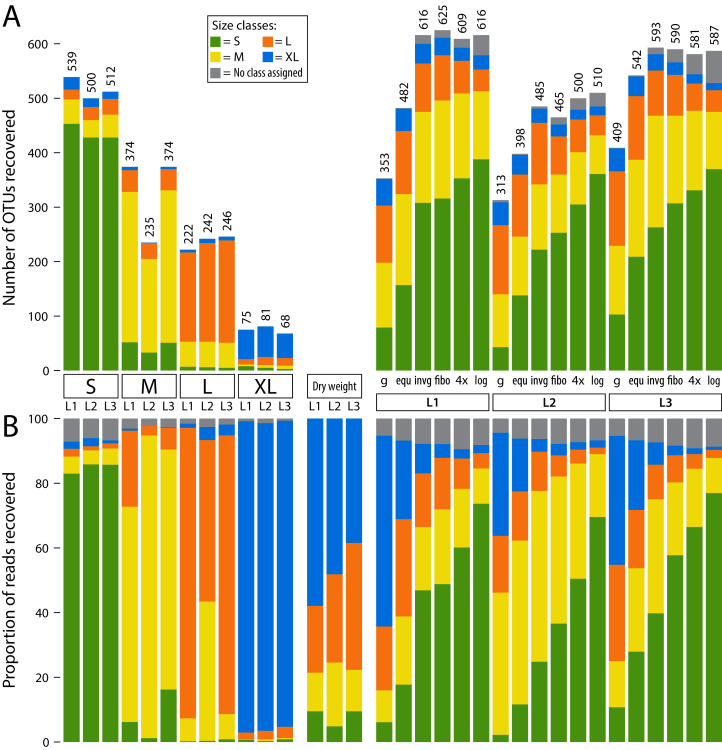
Detected OTUs across different samples (L1, L2 and L3). OTUs are assigned to size classes using the individually sequenced size fractions (S, M, L, XL). Some low-abundance OTUs were not recovered in individual size fractions, but in pooled samples. Thus, a size class was not assigned (grey bars). The four individual fractions were also pooled *in silico* at 1/4 sequencing depth (each filtered with a 0.04% threshold) to simulate equally pooling the samples. (A) barplots showing the number of OTUs recovered from individual size fractions as well as pooled samples (L1–L3). (B) barplots showing relative read abundance across samples (L1, L2 and L3). The “Dry weight” bars show the expected distribution of size classes based on relative abundance of dry weight of each size fraction, allowing a comparison to the proportionally pooled samples (g) that simulate a complete unsorted Malaise trap sample.

To test if the partitioning of a sample into two size fractions would be sufficient to achieve optimal taxon recovery, the individually sequenced size fractions S+M (S < 2 mm, M > 2 mm) and L+XL (L > 4 mm, XL > 8 mm) were combined and pooled *in silico* in different ratios (Fig. 2, Fig. S3). First, based on data obtained from the separately sequenced size fractions, sequences of class S and M were pooled *in silico* for each sample after quality filtering (sequences present in both replicates, representing at least 0.01% of total observed reads per sample). Pooling was conducted to simulate unsorted samples, according to the dry weight of each size fraction. An identical process was carried out for the sequences from size fractions L and XL, in which they were pooled to simulate unsorted samples (Fig. S3). In the next step, the two reconstructed fractions (S+M and L+XL) were pooled *in silico* in different proportions, based on a sequencing depth of 10,000 reads. Starting with 100% S+M and 0% L+XL, the ratio was altered in 5% steps to 0% S+M and 100% L+XL, taking into account read numbers (*e.g*. 10 × read abundance S+M, 0 × read abundance L+XL). Relative read numbers were calculated for merged samples and a filtering threshold of 0.01% was applied. Thereby OTUs with a read abundance <0.01% per sample were discarded. Detailed information about *in silico* pooling is available in Supplementary Material Script S2 (“silico_pool_v1.R”).

**Figure 3:**
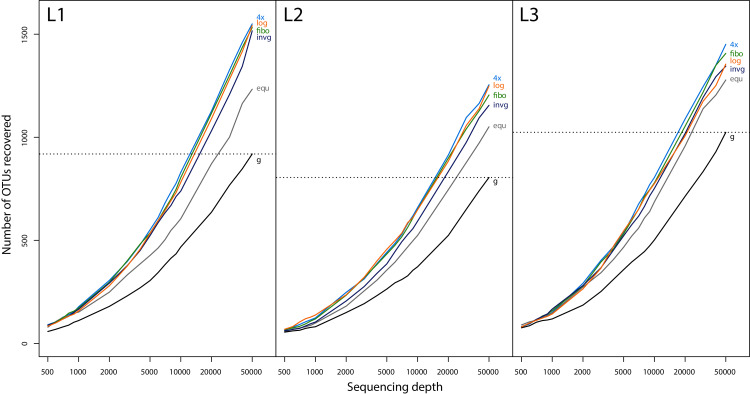
Rarefaction curves at different sequencing depths for all three samples (L1, L2 and L3) and lysates pooled in different proportions. The taxon recovery of the unsorted Malaise sample (g) at 50,000 reads sequencing depth is used to estimate the increase in sequencing depth needed to recover a similar number of OTUs to what is recovered from samples that are size sorted and pooled in favour of smaller size fractions. The OTU counts in this rarefaction analysis are slightly higher than in our other analyses since replicates were pooled directly, without removing reads that are not in both replicates and without discarding OTUs or reads below 0.01% abundance.

Rarefaction was applied to the unfiltered dataset (sequences present in both replicates, representing at least 0.01% of total observed reads per sample). The following examples detail a simulated sequencing depth of 500 reads (lowest sequencing depth) and 50,000 reads (highest sequencing depth (Fig. 3)). First, the sequencing depth was calculated using a filtering threshold defining the minimum abundance an OTU should have to be included in the analysis (for a sequencing depth of 500 reads: 100/500 = 0.2%; for a sequencing depth of 50,000 reads: 100/50,000 = 0.002%). In the next step, OTUs were excluded from the samples (g, equ, invg, fibo, log, 4×) if the minimum abundance of 0.2% (for 500 reads) or 0.002% (for 50,000 reads) was not reached respectively. Detailed information about *in silico* pooling is available in Supplementary Material Script S3 (“rarefaction.R”).

## Results

Sorting dried specimens into four size fractions using the “sieving tower” (Fig. S1A) took 2–3 h per sample in total as statically charged specimens and broken off tissue pieces had to be collected from the sieve surface. The alternative “shaker” design was also affected by electrostatic charging. This problem could be overcome by moistening the dried samples before transferring them, which however again results in a higher total processing time. Wet sieving was not affected by electrostatic charging of specimens and enabled size sorting in approximately 15 min including sample transfer. Alternating between gentle shaking and letting small specimens sink through the four mm mesh resulted in clear size fractionation with one order of magnitude difference in average specimen weight between the small and large size fractions (Fig. S1B).

The MiSeq run generated 21,911,822 PE reads which are available as raw data as well as demultiplexed samples under the NCBI SRA accession PRJNA625408. No-call events were observed on several tiles in read 2, leading to N being inserted at positions 16, 17 and 25. A total of 20 out of 38 tiles were affected by one or more No-call events, but as respective areas were mostly removed by primer trimming, analysis was not impaired. Average sequencing depth per sample was 249,423 reads (SD = 92.075) with the lowest sample containing 146,344 reads. In bioinformatic processing on average 23.40% (SD = 4.16%) of reads were discarded. After complete quality filtering, 99% of the 2,197 OTUs were assigned to the phylum Arthropoda (Table S2).

To assign a specimen size to each OTU, relative read abundance of all three samples was combined for each size class. Then, the size class with the highest abundance was selected as a specimen size estimate. Three quarters of OTUs were present in only one size fraction (Figs. S3, S4, S5). Thus, most OTUs could be clearly assigned to a size class using this strategy, with a few exceptions in the large and medium size category (*e.g*. OTU1 *Myrmica ruginodis* and OTU11 *Sphaerophoria*). Here, the size category with the highest read number was assigned, even when an OTU was found in high abundance in two fractions. For some low-abundance OTUs, no reads were assigned in any of the separately sequenced size classes, but only in the pooled samples (this was the case for 9.38% of OTUs). Thus, the respective OTUs had no size class assigned. Most OTUs were assigned to smaller size classes, with 41.51% in S, 24.53% in M, 19.21% in L and 5.37% in XL (Table S1). The individual size fractions (S, M, L and XL) recovered mostly reads from their respective size class (Fig. 2), with only a few exceptions (*e.g*. OUT1, *Myrmica ruginodis*). Here a high number of reads was assigned to size fraction L and M (43.2% and 55.7%). Thus, for OTU1 the size class M was assigned, despite the large size fraction also containing a substantial number of reads. The total number of taxa recovered was the highest in the smaller size fractions, and no more than 100 OTUs were recovered in the extra-large size fraction (Fig. 2).

When lysates were pooled based on dry size fraction weight (g) (simulating an unsorted Malaise trap sample), results were dominated by large specimens. Compared to the other pooling strategies, the simulated unsorted samples showed the weakest OTU recovery, followed by the pooling of all four lysates in equal proportions (equ). The other four pooling methods (invg, fibo, 4× and log) recovered more OTUs, with equally high numbers detected across the three Malaise trap samples. The logarithmic pooling (log) recovered the highest proportion of small specimens, while showing reduced recovery of large ones compared to the other methods. This was also supported by Jaccard and Bray–Curtis dissimilarity analysis (Fig. S6). Here, especially samples pooled using the log and 4× strategies cluster closely with samples where size class S had been sequenced individually. Samples pooled using strategies equ or g showed high similarity to size fraction L or even XL when read numbers were taken into account.

*In silico* pooling of the four individually sequenced size fractions at 1/4 sequencing depth of the lysates pooled in equal proportions (equ), showed that the individually sequenced size fractions recovered an average of 26.56% more OTUs than the equally pooled lysates (Fig. 2). However, the numbers of OTUs recovered from the *in silico* pool of individually sequenced size fractions were comparable to OTU counts recovered by lysates pooled in favour of small size fractions.

Rarefaction analysis showed that samples pooled in favour of small size fractions (invg, fibo, 4×, log) had a similar number of OTUs at around 15,000 reads sequencing depth compared to what was recovered from the unsorted sample (g) at 50,000 reads sequencing depth (Fig. 3). This represents a factor of approximately 3–4× reduction of sequencing depth compared to unsorted samples. In this comparison, unsorted samples (g) were more biased towards large specimens, while the samples pooled in favour of small specimens showed a higher detection rate of taxa assigned to the small size class (Fig. 2). Thus, while the number of OTUs recovered from the unsorted samples at 3–4× sequencing depth might be similar to that of size sorted samples, there are still differences in OTU composition between different pooling strategies (Fig. 2, Fig. S6).

To test if separation of a sample in only two size fractions would be sufficient to obtain optimal taxon recovery, individual size fractions S+M and L+XL were combined and pooled *in silico* in different ratios (Fig. S3). The recovery of OTUs was highest when adding 10–20% of the large fraction to the small fraction. The overall OTU recovery in the *in silico* pooled samples did in fact exceed the number of OTUs recovered by the processed lysate pools (invg, fibo, 4×, log) by up to 10–20%.

## Discussion

Previous studies have demonstrated that size sorting before metabarcoding can improve taxon recovery (Elbrecht, Peinert & Leese, 2017; Creedy, Ng & Vogler, 2019). This is particularly relevant when dealing with bulk samples containing specimens with a wide variation in biomass. We were able to confirm these trends for Malaise trap samples. Experiments were done on three Malaise samples only, which represents low replication effort and thus does not allow for statistical testing of results. However, the results presented here clearly support expectations and findings of previous studies (Elbrecht, Peinert & Leese, 2017; Creedy, Ng & Vogler, 2019). As illustrated in Figs. 2 and 3, our results present distinct patterns clearly showing differences between the applied strategies and therefore justifying the limited number of replicates and the absence of statistical testing.

Small and medium sized fractions contained the majority of OTUs in each of the three samples, consistent with studies from marine benthos (Wangensteen et al., 2018). When the four size fractions were pooled in different proportions, the fractions simulating a completely unsorted Malaise trap were dominated by large specimens, showing consistently the lowest recovery of OTUs. Interestingly, all four ways of pooling in favour of the smaller fractions (inverse weight, logarithmically, fibonacci based and 4-fold increase) recovered a similar number of OTUs. This result was unexpected, as for example logarithmic pooling strongly favours small fractions while severely underrepresenting larger ones. A reason for this effect could be OTU sharing across the individually sequenced size classes due to small specimens attached to larger ones, consequently also detected in larger fractions. This would also indicate that sieving does not perfectly separate individuals of different size classes. However, for the dry sieving approach, the aggregation of specimens of different size classes was rarely observed and counteracted through the shaking amplitude. The phenomenon occurred more often with wet sieving but was counteracted with an increased mesh surface, the use of higher ethanol volumes during sieving and a higher sieving amplitude. Furthermore, smaller specimens may be detected as secondary prey items in the guts of larger specimens and would therefore also be detected in larger size fractions. Additionally, legs and other body parts can easily break off the brittle specimens during dry sieving, and small size fractions therefore contain tissue from large specimens. Because small pieces of tissue will not break off from all specimens equally and large taxa found in the small fractions might not be representative of the real diversity in the samples, specimen damage during sieving and the subsequent detection of larger specimens in smaller size classes should be avoided. As shown here, wet sieving is a promising approach which is more gentle than dry sieving. In addition, different size fractions from different samples should be pooled in the same proportions for each sample to ensure comparability. Choice of pooling strategy might depend on study goals, and on size class and taxonomic composition of the samples, which can vary greatly depending on location. If taxa of interest fall within a particular size range, pooling should be adjusted accordingly, given that the number of reads and OTUs of differently sized specimens depends on the pooling process.

While we have shown that size sorting can increase taxon recovery in Malaise trap samples, it remains to be discussed how this can be done most efficiently. This is of particular importance for upscaled metabarcoding as required for biodiversity monitoring. Size sorting can only be used in larger scale metabarcoding projects if the process is time and cost efficient and does not substantially increase laboratory work. When the number of samples is limited and specimen biomass does not vary substantially, biomass biases can be mitigated (Creedy, Ng & Vogler, 2019) or minimized by only including a small part of the tissue (*e.g*. one leg) from large specimens (Ji et al., 2013; Carew, Coleman & Hoffmann, 2018). While manual sorting and processing of specimens can be a good strategy to reduce biomass bias, it is rather work intensive and difficult to scale or standardize. An automated approach would be desirable, but automated sieving techniques come with their own challenges. With the dry sieving approach used in this study, major methodological limitations arose, as it took several hours per sample to pick electrostatically charged individual specimens from the equipment. Consequently, we recommend using the alternative approach of wet sieving, completely immersing the specimens and sieve in ethanol. This approach can be automated more easily by stacking the sieve containers on shakers and thereby reducing hands-on time and increasing scalability. However, it needs to be considered that intensive cleaning of the equipment is required between sample processing steps to prevent cross contamination. Here, at least 15 min per sample should be calculated. Furthermore, wet sieving is conducted in pure 96% ethanol and the costs for additional amounts of ethanol need to be considered. Wet sieved specimens are less prone to damage and might be usable for morphological identification if non-destructive metabarcoding methods are applied, using for example preservative ethanol (Hajibabaei et al., 2012) or lysis buffer protocols (Nielsen et al., 2019).

Our *in silico* evaluations suggest that size sorting with a single sieve of four mm mesh size represents a good trade-off between taxon recovery and laboratory workload. Pooling the large and small size fractions in a proportion of 1:10 to 2:10 showed the highest taxon recovery in our *in silico* test, which however should be confirmed experimentally, since *in silico* comparisons can have limited reliability and due to the low replicate number processed in this study. For example, when *in silico* pooling 1/4 of each of the individually sequenced size fractions, more taxa were recovered than sequencing equally pooled lysate proportions. Thus, taxon recovery for the *in silico* estimates is slightly overrepresented, probably due to primer bias effects (Elbrecht & Leese, 2015; Piñol et al., 2015). For example, if a specific taxon in the large fraction is very well amplified, it will make up a large proportion of the reads in the pooled sample, decreasing the sequencing depth of all other specimens. When individual size fractions are sequenced, primer biases are limited to each individual fraction, increasing overall taxon recovery which is why some authors recommend the taxonomic sorting of bulk samples and sequencing each taxonomic group individually (Morinière et al., 2016; Beentjes et al., 2019). A taxonomic sorting step is however impractical for metabarcoding, as it substantially prolongs processing time per sample and does not necessarily increase taxon recovery as most arthropod primer binding sites show the same variability pattern (Elbrecht et al., 2019).

Other studies implement individual sequencing of each size fraction (Cowart et al., 2015; Wangensteen et al., 2018) and provided information about community composition for each size class to increase the detection of small and rare taxa. Sequencing of separate size classes doubles the costs for sample preparation and sequencing per sample. As an alternative, lysates or extracted DNA can be pooled proportionally to enrich the small size fractions sufficiently. Thereby, size sorting and subsequent pooling of lysates of each fraction before the DNA extraction is particularly advantageous as it reduces costs and time required per sample compared to pooling strategies after DNA extraction or PCR.

In our study, we confirm that size sorting can be applied to increase taxon recovery in metabarcoding of Malaise traps samples. However, the implementation of techniques, *e.g*. number of size fractions or pooling proportions, needs to be adjusted to the aim of the study. In some cases, size sorting may be skipped, as a sufficient signal can be achieved with a high sequencing depth for samples with limited specimen size variability (Creedy, Ng & Vogler, 2019). While Creedy, Ng & Vogler (2019) do not give exact numbers on sequencing depth effects, we simulate that a 3- to 4-fold increase in sequencing depth recovers similar numbers of OTUs from an unsorted sample compared to size sorted and proportionally pooled samples. Calculations are based on rarefaction analysis, which is explained in the materials and methods section (“*In silico* analysis”) and illustrated in Fig. 3. However, by increasing the sequencing depth, metabarcoding results will still be dominated by biomass rich taxa and it should be further experimentally tested how a higher sequencing depth can adequately replace size sorting.

Given that size sorting can be done in around 15 min using the wet sieving method demonstrated here, it seems like a worthwhile step to prepare Malaise trap samples for metabarcoding. Further Malaise trap sampling and additional size sorting experiments could be useful to quantify effects of large and abundant species on the detection of small or rare ones. Examination of Malaise trap samples or mock communities with known taxonomic composition and assigned size classes would be ideal to evaluate the results presented here. In addition to *in silico* assays, experimental approaches testing how a higher sequencing depth can compensate for size sorting and how different pooling proportions affect detected diversity patterns would be helpful to develop standardized monitoring protocols for flying arthropods.

## Conclusions

We demonstrate that size sorting of Malaise trap samples can increase recovery of small specimens. While our analyses show distinct patterns clearly supporting previous studies, the limited number of samples processed and the resulting lack of statistical support needs to be carefully considered. Pooling lysates in the extraction process can be an efficient way of improving the representation of small taxa. The methods presented here reduce laboratory work and costs, in comparison to sequencing each size fraction individually. *In silico* tests suggest that sieving into two size fractions (four mm mesh size) and subsequent pooling in a proportion of 1:10 (one part size class L and nine parts size class S) or 2:10 can substantially increase taxon recovery. Increasing sequencing depth by 3 to 4-fold can also be an alternative to size sorting, but might still underrepresent small and rare taxa due to biomass biases. Wet sieving of specimens in ethanol may be the best compromise for optimizing taxon recovery with low effort, compared to other approaches. However, this method remains to be thoroughly tested. Ultimately, the decision of whether or not samples should be size sorted depends on the research goals, budget and timeline as well as on the sample composition and size heterogeneity of specimens in the samples.

## Supplemental Information

10.7717/peerj.12177/supp-1 Supplemental Information 1 Overview of tested size sorting strategies. Overview of tested size sorting strategies. A: Dierent dry sieving strategies (”tower” and “shaker”, time consuming due to electrostatic charging of specimens) and wet sieving strategy in ethanol using a lunch box and a 4 mm metal mesh. B: Histogram showing the size and weight dierences of one additional malaise sample from Vlotho (52.122705N, 8.785186E) wet sieved into two size fractions using the “lunch box” design. Specimen body size refers to length measurements of specimens measured on milimeter scale paper without antennae or legs.

10.7717/peerj.12177/supp-2 Supplemental Information 2 Plate map for the DNA extraction from the 69 lysate samples. Plate map for the DNA extraction from the 69 lysate samples (including 9 negative controls), marked in green. Unmarked wells were part of another project sequenced on the same run. Additionally, the Illumina indexing used for PCR2 is indicated for each row (N702–N272) and column (S517–S503).

10.7717/peerj.12177/supp-3 Supplemental Information 3 OTU recovery for each sample. Barplot of OTU recovery for each sample (L1, L2, L3). Size fractions were sequenced individually. After bioinformatic analysis and quality ltering the fractions S and M as well as L and XL were pooled *in silico* according to dry weight of each sample fraction (simulate an unsorted sample). Next, the two fractions created (S+M and L+XL) were pooled *in silico* in dierent proportions that are illustrated on the x-axis of the graphs. Starting on the left side of the graph (proportion 1:20) one part of the larger size fraction (L+XL) was pooled with 19 parts of the smaller size fraction (S+M). Proportion change in 5% steps to the pool of 19 parts of the larger fraction (L+XL) with one part of the smaller fraction (S+M). Comparisons were done at 10,000 reads sequencing depth. Bars S+M and L+XL depict detected OTUs for only those two fractions at 10,000 reads sequencing depth.

10.7717/peerj.12177/supp-4 Supplemental Information 4 Overview of size fraction dry weight and OTU sharing between size fractions for all 3 malaise trap samples. A pie chart showing the dry specimen weight for each of the 4 individually sequenced size fractions. B UpSet plot (Lex 2014) showing the number of OTUs shared across the 4 size fractions for the 3 samples (L1, L2 and L3, in dierent shading).

10.7717/peerj.12177/supp-5 Supplemental Information 5 Heatmap indicating the relative read abundance for the dierent lysates. Replicates were merged, and reads only present in one sample discarded. Reads are subsampled to 10,000 reads per sample (reads with abundance below 0.01% were discarded). See Table S2 for the raw data.

10.7717/peerj.12177/supp-6 Supplemental Information 6 Dendrogram of dissimilarity indices. Dendrogram of dissimilarity indices including A) Jaccard and B) Bray–Curtis (right) dissimilarity. Both analysis include separated sequenced size classes of the three samples (L1–L3) and samples processed in dierent pooling strategies (g, log10, invg, bo, equ, 4x).

10.7717/peerj.12177/supp-7 Supplemental Information 7 Volumes of pooled lysate for different pooling strategies.

10.7717/peerj.12177/supp-8 Supplemental Information 8 OTU table of separately sequenced size classes as well as different pooling strategies.

10.7717/peerj.12177/supp-9 Supplemental Information 9 JAMP script for data analysis.

10.7717/peerj.12177/supp-10 Supplemental Information 10 R script for *in silico* analysis.

10.7717/peerj.12177/supp-11 Supplemental Information 11 Procedure of rarefaction analysis illustrated in Figure 3.

## Additional Information and Declarations

### Field Study Permissions

The following information was supplied relating to field study approvals (*i.e*., approving body and any reference numbers): Field experiments were approved by the nature conservation authority Euskirchen (reference number: 67.64.300/Pa).

### Data Availability

The following information was supplied regarding data availability: The raw sequence used in this study is available at NCBI SRA: PRJNA625408.
