# Supplementary material for: Pooling size sorted Malaise trap fractions to maximize taxon recovery with metabarcoding
Source: PeerJ. 2021 Oct 5;9:e12177. doi: 10.7717/peerj.12177 (PMC8500090; doi:10.7717/peerj.12177)
Supplement: Supplemental Information 1 — Overview of tested size sorting strategies. A: Dierent dry sieving strategies (”tower” and “shaker”, time consuming due to electrostatic charging of specimens) and wet sieving strategy in ethanol using a lunch box and a 4 mm metal mesh. B: Histogram showing the size and weight dierences of one additional malaise sample from Vlotho (52.122705N, 8.785186E) wet sieved into two size fractions using the “lunch box” design. Specimen body size refers to length measurements of specimens measured on milimeter scale paper without antennae or legs. [file peerj-09-12177-s001.pdf]

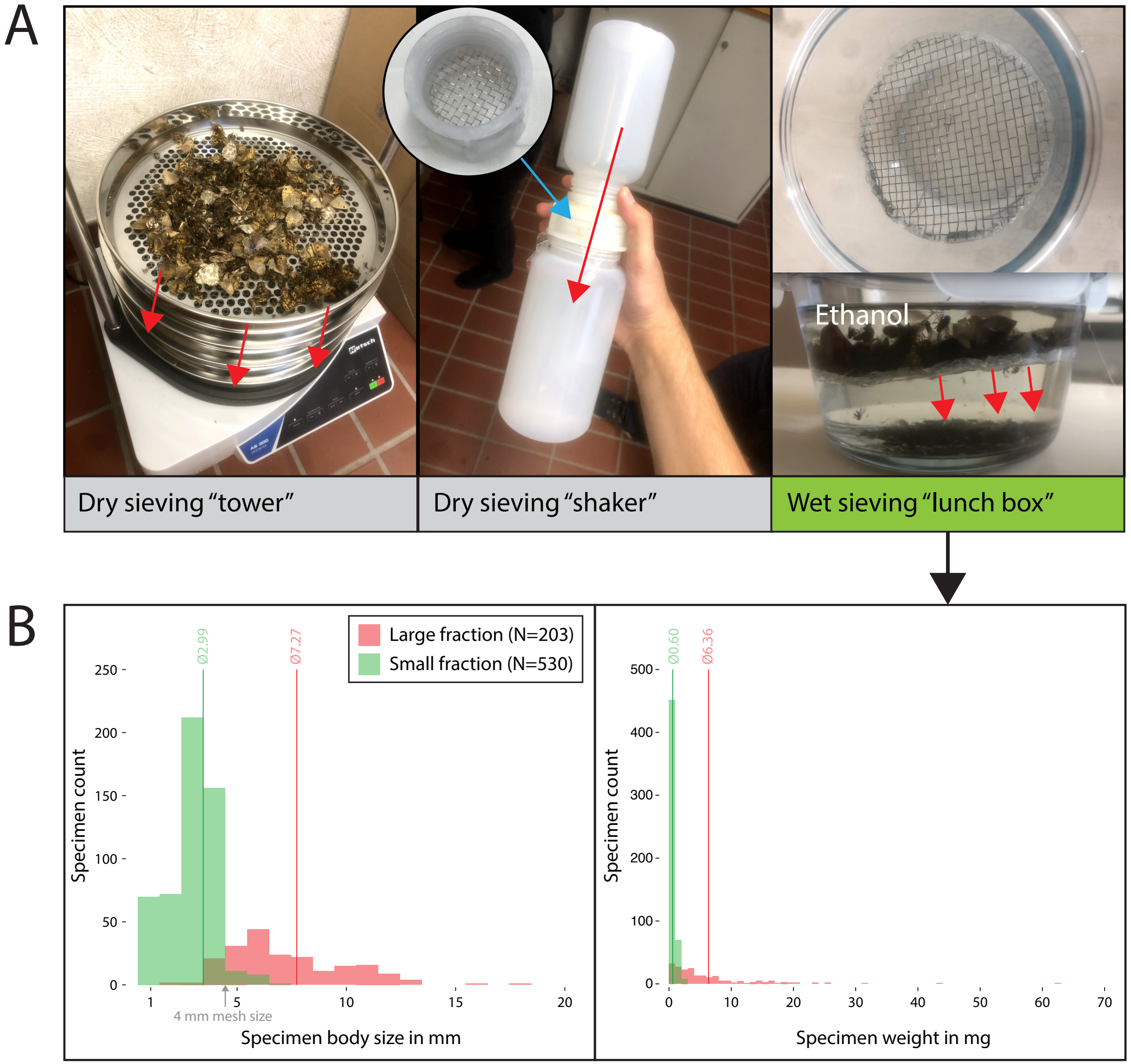

Fig. S1: Overview of tested size sorting strategies. A: Different dry sieving strategies (”tower” and “shaker”, time consuming due to electrostatic charging of specimens) and wet sieving strategy in ethanol using a lunch box and a 4 mm metal mesh. B: Histogram showing the size and weight differences of one additional malaise sample from Vlotho (52.122705N, 8.785186E) wet sieved into two size fractions using the “lunch box” design. Specimen body size refers to length measurements of specimens measured on milimeter scale paper without antennae or legs.
