# Supplementary material for: Pooling size sorted Malaise trap fractions to maximize taxon recovery with metabarcoding
Source: PeerJ. 2021 Oct 5;9:e12177. doi: 10.7717/peerj.12177 (PMC8500090; doi:10.7717/peerj.12177)
Supplement: Supplemental Information 2 — Plate map for the DNA extraction from the 69 lysate samples (including 9 negative controls), marked in green. Unmarked wells were part of another project sequenced on the same run. Additionally, the Illumina indexing used for PCR2 is indicated for each row (N702–N272) and column (S517–S503). [file peerj-09-12177-s002.pdf]

index 2: / index 1:	S517<br>H	S511<br>G	S510<br>F	S508<br>E	S507<br>D	S506<br>C	S505<br>B	S503<br>A
N702 1	D<br>C01	D<br>L1_MA	D<br>L1_XLB	D<br>L2_fiboA	D<br>L2_equB	D<br>L2_SB	D<br>L3_XLA	D<br>L3_logB
N703 2	D<br>L1_gA	D<br>L1_SA	D<br>L1_LB	D<br>L2_4xA	D<br>L2_logB	D<br>L3_gA	D<br>L3_LA	D<br>C2
N704 3	D<br>L1_invgA	D<br>L1_gB	D<br>L1_MB	D<br>C03	D<br>L2_fiboB	D<br>L3_invgA	D<br>L3_MA	D<br>L3_fiboB
N705 4	D<br>L1_equA	D<br>L1_invgB	D<br>L1_SB	D<br>L2_XLA	D<br>L2_4xB	D<br>C4	D<br>L3_SA	D<br>L3_4xB
N711 5	D<br>L1_logA	D<br>C05	D<br>L2_gA	D<br>L2_LA	D<br>L2_XLB	D<br>L3_equA	D<br>L3_gB	D<br>L3_XLB
N712 6	D<br>L1_fiboA	D<br>L1_equB	D<br>L2_invgA	D<br>L2_MA	D<br>C6	D<br>L3_logA	D<br>L3_invgB	D<br>L3_LB
N714 7	D<br>L1_4xA	D<br>L1_logB	D<br>C07	D<br>L2_SA	D<br>L2_LB	D<br>L3_fiboA	D<br>L3_equB	D<br>L3_MB
N715 8	D<br>L1_XLA	D<br>L1_fiboB	D<br>L2_equA	D<br>L2_gB	D<br>L2_MB	D<br>L3_4xA	D<br>C8	D<br>L3_SB
N718 9	D<br>L1_LA	D<br>L1_4xB	D<br>L2_logA	D<br>L2_invgB	D<br>C9	C<br>101<br>101_3	C<br>Kit_M<br>Kit_M3	D<br>118abd<br>Abd_mock4
N723 10	A<br>A_M<br>A_M1	A<br>103<br>103_1	A<br>Kit_M<br>Kit_M1	B<br>103<br>103_2	B<br>44.1<br>GBOL_mock2	C<br>103<br>103_3	D<br>A_M<br>A_M4	C<br>C10
N724 11	A<br>C11	A<br>118abd<br>Abd_mock1	B<br>A_M<br>A_M2	B<br>118abd<br>Abd_mock2	B<br>Kit_M<br>Kit_M2	C<br>118abd<br>Abd_mock3	D<br>101<br>101_4	D<br>44.1<br>GBOL_mock4
N727 12	A<br>101<br>101_1	A<br>44.1<br>GBOL_mock1	B<br>101<br>101_2	B<br>C12	C<br>A_M<br>A_M3	C<br>44.1<br>GBOL_mock3	D<br>103<br>103_4	D<br>Kit_M<br>Kit_M4

Fig. S2: Plate map for the DNA extraction from the 69 lysate samples (including 9 negative controls), marked in green. Unmarked wells were part of another project sequenced on the same run. Additionally, the Illumina indexing used for PCR2 is indicated for each row (N702-N272) and column (S517-S503).
