# Supplementary material for: Pooling size sorted Malaise trap fractions to maximize taxon recovery with metabarcoding
Source: PeerJ. 2021 Oct 5;9:e12177. doi: 10.7717/peerj.12177 (PMC8500090; doi:10.7717/peerj.12177)
Supplement: Supplemental Information 3 — Barplot of OTU recovery for each sample (L1, L2, L3). Size fractions were sequenced individually. After bioinformatic analysis and quality ltering the fractions S and M as well as L and XL were pooled in silico according to dry weight of each sample fraction (simulate an unsorted sample). Next, the two fractions created (S+M and L+XL) were pooled in silico in dierent proportions that are illustrated on the x-axis of the graphs. Starting on the left side of the graph (proportion 1:20) one part of the larger size fraction (L+XL) was pooled with 19 parts of the smaller size fraction (S+M). Proportion change in 5% steps to the pool of 19 parts of the larger fraction (L+XL) with one part of the smaller fraction (S+M). Comparisons were done at 10,000 reads sequencing depth. Bars S+M and L+XL depict detected OTUs for only those two fractions at 10,000 reads sequencing depth. [file peerj-09-12177-s003.pdf]

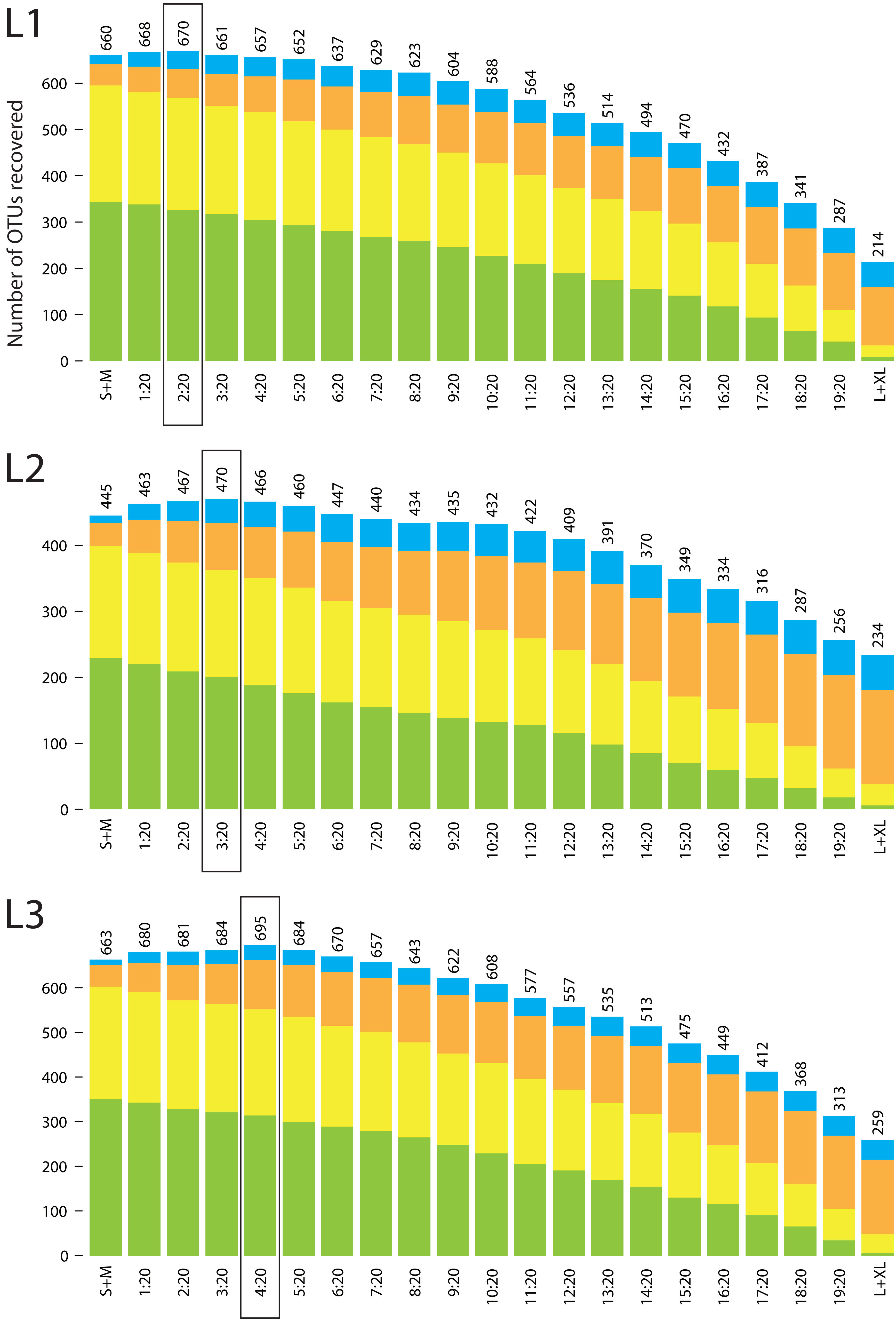

Fig. S3: Barplot of OTU recovery for each sample (L1, L2, L3). Size fractions were sequenced individually. After bioinformatic analysis and quality filtering the fractions S and M as well as L and XL were pooled in silico according to dry weight of each sample fraction (simulate an unsorted sample). Next, the two fractions created (S+M and L+XL) were pooled in silico in different proportions that are illustrated on the x-axis of the graphs. Starting on the left side of the graph (proportion 1:20) one part of the larger size fraction (L+XL) was pooled with 19 parts of the smaller size fraction (S+M). Proportion change in 5% steps to the pool of 19 parts of the larger fraction (L+XL) with one part of the smaller fraction (S+M). Comparisons were done at 10,000 reads sequencing depth. Bars S+M and L+XL depict detected OTUs for only those two fractions at 10,000 reads sequencing depth.
