# Supplementary material for: Pooling size sorted Malaise trap fractions to maximize taxon recovery with metabarcoding
Source: PeerJ. 2021 Oct 5;9:e12177. doi: 10.7717/peerj.12177 (PMC8500090; doi:10.7717/peerj.12177)
Supplement: Supplemental Information 4 — A pie chart showing the dry specimen weight for each of the 4 individually sequenced size fractions. B UpSet plot (Lex 2014) showing the number of OTUs shared across the 4 size fractions for the 3 samples (L1, L2 and L3, in dierent shading). [file peerj-09-12177-s004.pdf]

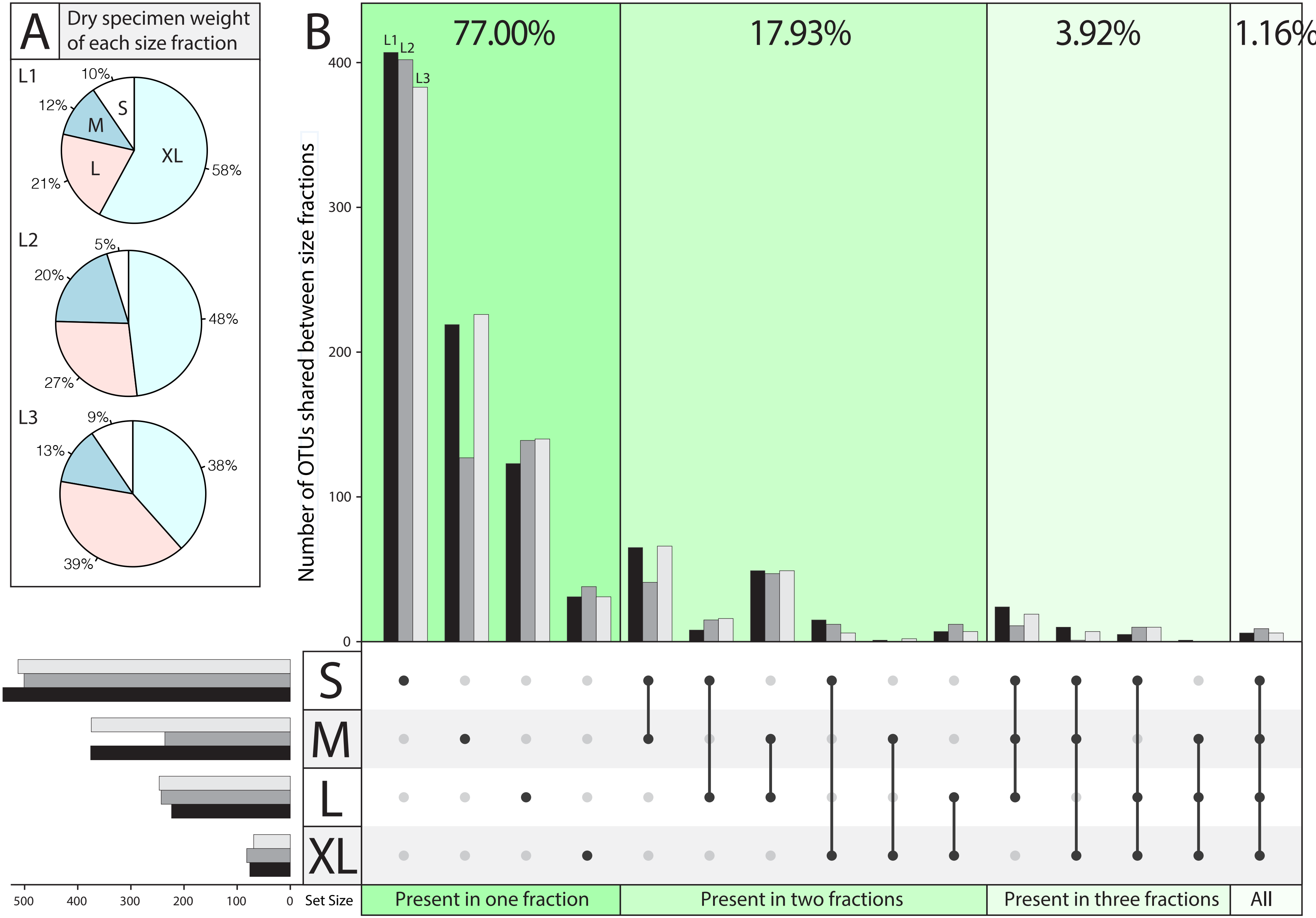

Fig. S4: Overview of size fraction dry weight and OTU sharing between size fractions for all 3 malaise trap samples. A Pie charts showing the dry specimen weight for each of the 4 individually sequenced size fractions. B UpSet plot (Lex 2014) showing the number of OTUs shared across the 4 size fractions for the 3 samples (L1, L2 and L3, in different shading).
