# Supplementary material for: Pooling size sorted Malaise trap fractions to maximize taxon recovery with metabarcoding
Source: PeerJ. 2021 Oct 5;9:e12177. doi: 10.7717/peerj.12177 (PMC8500090; doi:10.7717/peerj.12177)
Supplement: Supplemental Information 6 — Dendrogram of dissimilarity indices including A) Jaccard and B) Bray–Curtis (right) dissimilarity. Both analysis include separated sequenced size classes of the three samples (L1–L3) and samples processed in dierent pooling strategies (g, log10, invg, bo, equ, 4x). [file peerj-09-12177-s006.pdf]

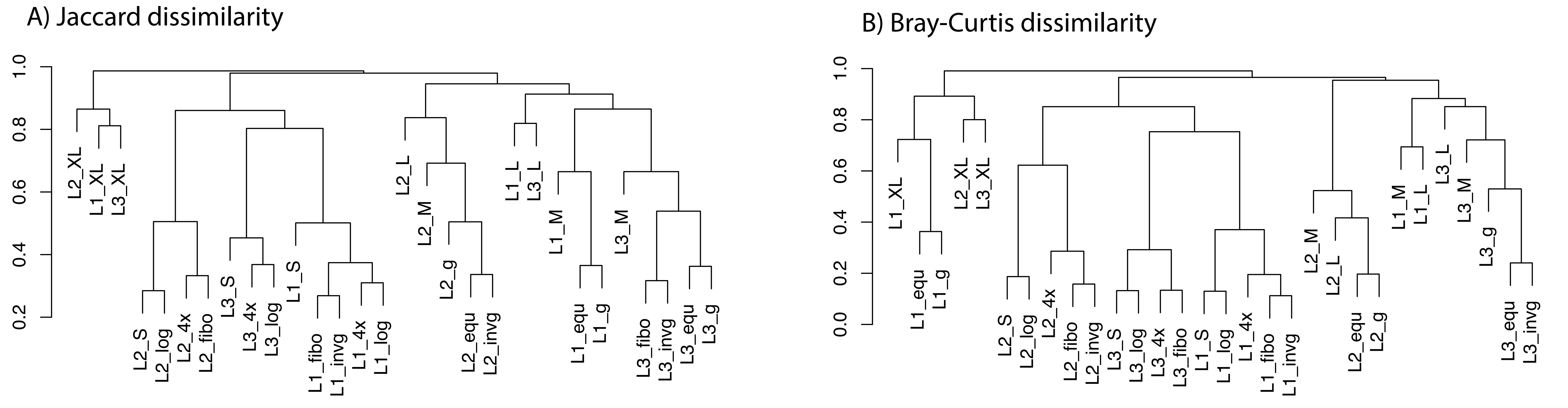

Fig. S6: Dendrogram of dissimilarity indices including A) Jaccard and B) Bray-Curtis (right) dissimilarity. Both analysis include separated sequenced size classes of the three samples (L1-L3) and samples processed in different pooling strategies (g, log10, invg, fibo, equ, 4x).
